# Knowledge and practice of essential newborn care and associated factors among women in Ethiopia: systematic review and meta-analysis

**DOI:** 10.1186/s12978-022-01480-0

**Published:** 2022-08-04

**Authors:** Alemu Degu Ayele, Lebeza Alemu Tenaw, Bekalu Getnet Kassa, Gedefaye Nibret Mihretie, Habtamu Gebrehana Belay, Adanech Getie Teffera, Eden Workneh Aychew, Enyew Dagnew Yehuala, Tigist Seid Yimer

**Affiliations:** 1 Department of Midwifery, College of Health Sciences, Debre Tabor University, Debre Tabor, Ethiopia; 2 School of Public Health, College of Health Sciences, Woldia University, Woldia, Ethiopia

**Keywords:** Newborn, Essential, Knowledge, Practice, Ethiopia

## Abstract

**Background:**

In developing countries, including Ethiopia the risk of neonatal death can be easily prevented and avoided by implementing essential newborn care with simple, low cost, and a short period time immediately after delivery. However, the problem is still persisting due to lack of adequate maternal and newborn care practice. Hence, this review aimed to estimate the pooled prevalence of women’s knowledge and practice of essential newborn care and its associated factors in Ethiopia using systematic review and meta-analysis.

**Method:**

An intensive literature search was performed from PubMed, Google Scholar, EMBASE, HINARI, Scopus, and Web of Sciences from April 1–30, 2021. Data were extracted by using a pre-tested and standardized data extraction format. The data were analyzed by using STATA 14 statistical software. I^2^ tests assessed heterogeneity across the included studies. A random-effect model was used to estimate the pooled prevalence of knowledge and practice of essential newborn care.

**Results:**

From 1275 identified studies, 25 articles were included. The national pooled prevalence of essential newborn care knowledge and practice among women was 55.05% and 41.49% respectively. Secondary education (AOR = 2.75, 95% CI 1.62, 4.66), multiparity (AOR = 2.14, 95% CI 1.41, 3.26), antenatal care (AOR = 2.94; 95% CI 2.03, 4.26), and postnatal follow-up (AOR = 1.64, 95% CI 1.20, 2.23) were significantly associated with knowledge level whereas; primary education (AOR = 7.08, 95% CI 4.79, 10.47), urban residency (AOR = 2.22, 95% CI 1.65, 3.00), attending monthly meetings (AOR = 2.07, 95% CI 1.64, 2.62), antenatal care (AOR = 2.89, 95% CI 1.97, 4.26), advised during delivery (AOR = 2.54, 95% CI 1.80, 3.59), postnatal follow-up (AOR = 7.08, 95% CI 4.79, 10.47) and knowledge (AOR = 2.93; 95% CI 1.81, 4.75) were statistically significant with essential newborn practice.

**Conclusions:**

The current systematic review and meta-analysis findings reported that the level of knowledge and practice of essential newborn care among Ethiopian women was low. Therefore, improvement of essential newborn through the provision of community-based awareness creation forum, improving antenatal and postnatal care follow up, education on essential newborn care to all pregnant and postnatal women are very important.

*Trial registration* Prospero registration: CRD 42021251521

**Supplementary Information:**

The online version contains supplementary material available at 10.1186/s12978-022-01480-0.

## Plain Language Summary

Essential newborn care (ENC) is a single most cost-effective intervention to reduce neonatal mortality and morbidity both in developed and developing countries. An intensive electronic search from PubMed, Google Scholar, EMBASE, HINAR, Scopus, and Web of Sciences were performed to identify the primary studies. In this meta-analysis a total of 25 articles were included to estimate the polled prevalence of ENC knowledge and practice among Ethiopian women. Accordingly, the national pooled prevalence of ENC knowledge and practice among women was 55.05% and 41.49% respectively. In the current systematic review and meta-analysis, Secondary education, multiparity, antenatal care, and postnatal follow-up were significantly associated with knowledge level whereas; primary education, urban residency, attending monthly meetings, antenatal care, advised during delivery, postnatal follow-up and knowledge on ENC were found to be statistically significant with essential newborn practice. As per finding the knowledge and practice regarding essential newborn care among women in Ethiopian was significantly low. Hence, improvement of essential newborn through the provision of community-based awareness creation forum, improving antenatal and postnatal care follow up, education on essential newborn care to all pregnant and postnatal women are very important.

**Supplementary Information:**

The online version contains supplementary material available at 10.1186/s12978-022-01480-0.

## Introduction

Since maternal health and newborn health are inextricably linked; obstetric care provided during the perinatal and neonatal periods is critical to ensuring the health of the mother and baby [1].

Neonatal mortality rates are basic indicator of a countries socioeconomic status and quality of health care [2]. As the first 28 days of neonatal periods are the most venerable time for infant death, newborn during this period needs a careful attention to improve their survival [3].

Although the neonatal period is a brief and short period time, neonatal mortality accounts for approximately two-thirds of all infant mortality and 45% of under-five mortality in the globe [4]. Globally almost all neonatal deaths (98%) occur in low- and middle-income countries like Ethiopia with half of deliveries occurring at home [5–7].

Essential newborn care (ENC) is a comprehensive strategy designed to improve the health of newborns through interventions before conception, during pregnancy, during delivery, and the immediate postnatal period. It is a single most cost-effective intervention to reduce neonatal mortality and morbidity both in developed and developing countries [1, 8].

WHO has formulated a set of guidelines about the Essential Newborn care Practices (ENCP) which are evidence-based and cost-effective measures to improve neonatal health outcomes. This guideline is to be used by all stakeholders who engaged with the neonate including the health care providers, mother, community, and government [9]. The mother should have to begin breast feeding as soon as possible within an hour following giving birth [10].

ENC comprises: basic preventive newborn care such as (care before and during pregnancy, clean delivery practices, temperature maintenance, eye and cord care, and early and exclusive breastfeeding and early detection of problems or danger signs and appropriate referral and care seeking [1].

In developing countries, the risk of neonatal death can be easily prevented and avoided by implementing essential newborn care with simple, low cost, and a short period time; however, the problem is still persisting due to lack of adequate maternal neonatal care practices [11–14].

The global society had set a sustainable developmental goal (SDG) aiming to reduce the neonatal and under-five mortality rate to 12 and 25 deaths per 1000 live birth respectively at 2030 [15]. With this regard Ethiopia has formulating the community-based newborn care (CBNC) implementation programs in 2013 to improve community-based maternal and newborn health service, including the provision of immediate newborn care, initial stimulation and resuscitation of the newborn, prevention and management of hypothermia, management of pre-term and low birth weight (LBW) neonates, and management of neonatal sepsis and very severe disease at the community level [16].

However, the burden of neonatal mortality is still remains as an unaccomplished agenda [17]. The Mini- Ethiopian demographic and Health Survey (EDHS) 2019 report showed, that the rate of neonatal, infant and under-five mortality was 30%, 43% and 55% per 1000 live births respectively [18].

Numerous primary studies conducted in Ethiopia tried to estimates women’s knowledge and practice of essential newborn care [19–43]. According to the findings, the knowledge of women on ENC ranged from 36.1% [25] to 80.4% [21]. The practice of ENC among women also ranged from 13.7% [33] to 81.1% [25]. According to the findings there is a significant variation between the level of knowledge and practice of ENC among women at the national level.

The reason for these discrepancy in the level of knowledge and practice of ENC among Ethiopian women to date was not investigated. Hence, it is essential to estimate the pooled prevalence of ENC knowledge and practice and determinant factors to identify existing gaps. The finding of this review also provides recommendations regarding strategies that scale up the neonatal, infant, under-five, and at large the health of the community in Ethiopia.

## Methods

### Study design and protocol registration

This was a systemic review and meta-analysis of pertinent observational studies on knowledge and practice of ENC among women in Ethiopia. The protocol has been registered on an International Prospective Register of Systematic Review (PROSPERO, number CRD 42021251521) and performed following the Preferred Reporting Items for Systematic Reviews and Meta-Analyses (PRISMA) checklist) [44].

### Eligibility criteria

#### Inclusion criteria

Study area: Studies reporting the prevalence of knowledge and practice of ENC and predictors among women in Ethiopia.

Study design: Observational (cross-sectional) studies that measured the prevalence of knowledge and practice of ENC among women in Ethiopia.

Publication status: All published studies and studies found on websites of Ethiopian universities and research institutes.

Language: Articles written only in the English language were considered.

Population: Women who had newborns and resided in Ethiopia.

Publication year: All research reports from January 2010 until April 31, 202, were incorporated.

Exposure: Predictors/determinants of ENC. The determinants are factors that increase or decrease the likelihood of ENC knowledge and practice.

Outcome: Women who are knowledgeable on ENC and properly practice it.

#### Exclusion criteria

Studies that were not published in English and unable to access a copy translated into the English language were excluded. Similarly, pure qualitative studies assessed ENC knowledge and practice, reviews, essays, conference abstracts, letters, and commentaries were excluded from the study. Besides, when we encounter duplicated publications from the same study population within the same year, we exclude the publication that had incomplete information of the results.

Finally, non-accessible studies which were unpublished, irretrievable from the databases or failed to receive replies to an email from corresponding authors were excluded.

### Searching strategy and data source

An intensive literature search was performed from PubMed, Google Scholar, EMBASE, HINAR, Scopus, and Web of Sciences from April 1–30, 2021. Besides, Grey literature deposited at universities and research institutes’ websites online repository were also searched. Moreover, reference lists of screened studies were checked. All studies that reported ENC women’s knowledge and practice of ENC in Ethiopia from January 2010 to April 31, 2021, were included in the review. Initially, studies were searched by examining the full titles (“Knowledge and practice of essential newborn care and associated factors among women in Ethiopia”) and then by using the following terms and phrases (“Knowledge” AND “practice” AND “essential” AND “newborn” AND “care” AND “associated factors” OR “predictors” OR “determinants” AND “women” AND “Ethiopia”) (Additional file 1).

### Identification and study selection

All searched studies were exported to the Endnote X7 reference manager software, and duplicated articles were excluded. Studies were screened after reading the title and abstracts. Three authors (AD, BG, and HG) screened and assessed the studies independently. The studies full text was further assessed based on aims, methodology, participants/population, and critical findings (knowledge and practice of ENC). Any disagreements between the three authors were resolved through discussion and consensus based on established criteria or through two investigators (AG and EW).

### Quality assessment

The qualities of all included articles were assessed for overall inclusion and evidence synthesis in the analysis using the Newcastle–Ottawa scale (NOS) quality assessment tool adapted for cross-sectional studies quality assessments [45]. The tool consists of ten items (stars) divided into three core components. The first component of the tool is rated from five stars and mainly focuses on the methodological quality of each primary study (i.e., sample size, response rate, sampling technique, and ascertainment of the exposure or risk factor). The second component of the tool emphasized the comparability of the study (study controls for the most important factor and study control for any additional factor) and rated as two stars. The tool’s last section assessed the quality of primary articles in statistical analysis and outcome point of view and was based on three stars. Four authors (AG, EW, ED, and TS) assessed the quality of each primary article thoroughly and independently using the above pointers. Any disagreements between the three reviewers were discussed until a consensus was reached if not managed by taking the average score of their critical appraisal outcomes. Finally, those articles with a mean quality score of ≥ 7 out of 10 were undertaken for analysis (Additional file 2).

### Data extraction

Data were extracted from primary articles included in the review by three of the authors (AD, LA, and BG) using a pre-tested and standardized data extraction format. The data extraction format includes, first author, publication year, study regions, study setting, study population, sample size, response rate, the prevalence of ENC knowledge and practice, and determinants of ENC knowledge and practice with a 95% confidence interval.

### Outcome measures

Women’s level of knowledge and practice of ENC were the principal outcomes reported in this systematic review and meta-analysis. The second objective of this review was to identify factors associated with the level of knowledge and practice of ENC.

### Heterogeneity and publication bias

The presence of heterogeneity (variation) between the included studies was assessed by calculating the I^2^ statistics and its corresponding p-value. I^2^ statistics of 25%, 50%, and 75% were used to declare low, moderate, and high heterogeneity, respectively [46]. To identify any possible source of heterogeneity subgroup analysis was conducted by regions and study setting. Besides, sensitivity analysis was carried out to identify any outlier studies.

For the valuation of the publication bias of the included studies, we used a visual inspection of the funnel plot graph qualitatively. We also objectively measure publication bias by computing Egger’s regression test and p < 0.05 declared presence of publication bias [47, 48].

### Statistical analysis

Extracted data were entered using Microsoft Excel and exported to STATA version 14 software for further analysis. The associated factors of ENC were examined based on eligibility criteria. We had considered at least two studies that reported on one associated factor of ENC in common with their measure of effect and 95% confidence interval (CI). The random effects model based on the DerSimonian–Laird method was considered to assess for variations between the studies. The results were presented using texts, tables, and forest plots with measures of effect and a 95% confidence interval.

## Results

### Study identification

**Fig. 1:**
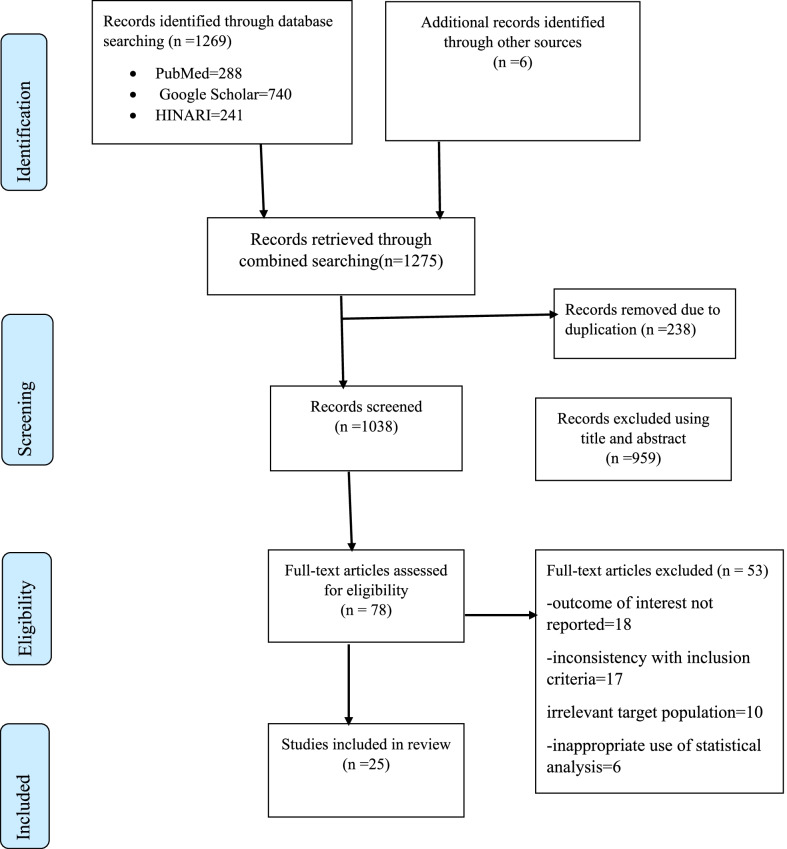
Flow chart of study selection for systematic review and meta-analysis of knowledge and practice of essential newborn care among women in Ethiopia

A total of 1275 recorded articles were identified using an electronic database. Of these records, 238 studies were excluded due to duplication. Out of the remaining 1037 studies, 959 studies were excluded after reviewing of title and abstract since they are unrelated to the title. The remaining 78 full-text articles were further accessed and screened based on the pre-set criteria for potential inclusion. Of these, 53 articles were excluded due to the outcome of interest not reported, inconsistent with inclusion criteria, unrelated target population, and inappropriate use of statical analysis. Finally, 25 studies were found to be eligible and included in the systematic review and meta-analysis (Fig. 1).

### Study characteristics

All the 25 included studies used cross-sectional study design and reported in the English language. Out of the 25 articles that were eligible and included in this meta-analysis, eight studies were conducted in Southern Nation Nationalities and Peoples Region (SNNPR) [22, 23, 26, 31, 34, 35, 39, 42], six studies conducted in Amhara region [20, 32, 33, 36, 38, 43], five studies in Tigray regional state [21, 25, 29, 30, 37], two from Oromia region [28, 41], two from Addis Ababa (capital city of Ethiopia) [19, 27], and one study conducted in Benishangul Gumz regional state of Ethiopia [40], and one from Harari [24]. In the current systematic review and meta-analysis, a total of participants was involved 13,001 with a response rate ranged from 96.6 to 100%. The sample size of studies ranged from a minimum of 186 women [43] to a maximum of 970 women in Amhara [33]. Twenty-one of the included studies reported on the practice of ENC [20, 22, 23, 25, 27–43], and nine provided data on knowledge of ENC [19–26, 43] (Table 1). From the 25 included studies, nine studies had a quality score of seven, eight studies had a quality score of eight, and the remaining eight had a quality score of nine. Hence, all of them had a good and above quality score (Additional file 2).

**Table 1:** Summary of the 25 primary studies included in the systematic review and meta-analysis assessing women level of knowledge and practice of essential newborn care in Ethiopia

Author’s, year	Region	Study design	Study setting	Sample size	R. rate (%)	Parameter studied	Prevalence (%) with 95% CI
Berhan et al. [19]	A. A	C/S	Institution	512	100	Knowledge	39.8 (37.66, 41.94)
Kebede [20]	Amhara	C/S	Institution	414	98.1	Knowledge and practice	55.30 (53.13, 57.47)
							60.60 (58.47, 62.73)
Misgna et al. [21]	Tigray	C/S	Community	296	100	Knowledge	80.40 (78.67, 82.13)
Abebe et al. [22]	SNNPR	C/S	Community	624	97.4	Knowledge and practice	50.00 (47.82, 52.18)
							41.00 (38.86, 43.14)
Thomas and Funga [23]	SNNPR	C/S	Community	399	97	Knowledge and practice	37.70 (35.59, 39.81)
							34.10 (32.03, 36.17)
Zewde [24]	Harar	C/S	Institutional	266	96.6	Knowledge	57.20 (55.04, 59.36)
Berhea et al. [25]	Tigray	C/S	Community	456	100	Knowledge and practice	36.10 (34.00, 38.20)
							81.10 (79.39, 82.81)
Mersha et al. [26]	SNNPR	C/S	Community	630	100	Knowledge	57.60 (55.44, 59.76)
Workinesh et al. [27]	A. A	C/S	Institution	576	100	Practice	38.80 (36.67, 40.93)
Derese et al. [28]	Oromia	C/S	Community	632	98.1	Practice	18.90 (17.19, 20.61)
Teferi et al. [29]	Tigray	C/S	Community	389	100	Practice	78.90 (77.12, 80.68)
Berhe et al. [30]	Tigray	C/S	Institutional	423	100	Practice	26.70 (24.77, 28.63)
Agonafir et al. [31]	SNNPR	C/S	Community	490	100	Practice	29.00 (27.02, 30.98)
Kokebie [32]	Amhara	C/S	Community	570	97.01	Practice	23.10 (21.26, 24.94)
Tafere et al. [33]	Amhara	C/S	Institution	970	84.8	Practice	13.70 (12.20, 15.20)
Mersha et al. [34]	SNNPR	C/S	Community	630	100	Practice	38.40 (36.28, 40.52)
Chichiabellu [35]	SNNPR	C/S	Community	450	100	Practice	24.00 (22.14, 25.86)
Semanew et al. [36]	Amhara	C/S	Institution	423	98.8	Practice	46.90 (44.72, 49.08)
Weldeargeawi et al. [37]	Tigray	C/S	Community	634	100	Practice	40.70 (38.56, 42.84)
Asnakew et al. [38]	Amhara	C/S	Community	814	100	Practice	45.80 (43.63, 47.97)
Alemu et al. [39]	SNNPR	C/S	Community	834	100	Practice	24.10 (22.23, 25.97)
Tegene et al. [40]	Benishangul	C/S	Community	539	98.7	Practice	40.60 (38.46, 42.74)
Efa et al. [41]	Oromia	C/S	Institution	422	98.8	Practice	44.10 (41.93, 46.27)
Sakelo et al. [42]	SNNP	C/S	Community	422	100	Practice	31.00 (28.98, 33.02)
Yisak et al. [43]	Amhara		Institution	186	100	Knowledge and practice	81.20 (79.50, 82.90)
							89.90 (88.48, 91.12)

### Women’s knowledge and practice of ENC in Ethiopia

**Fig. 2:**
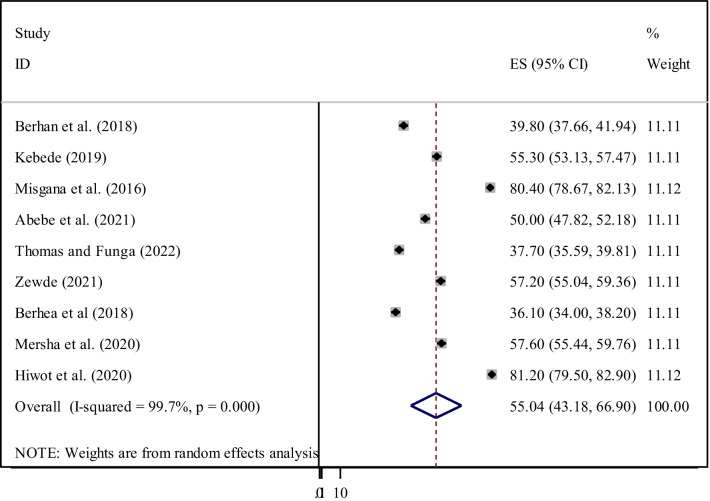
Forest plot of the pooled prevalence of women’s knowledge on essential newborn care in Ethiopia

**Fig. 3:**
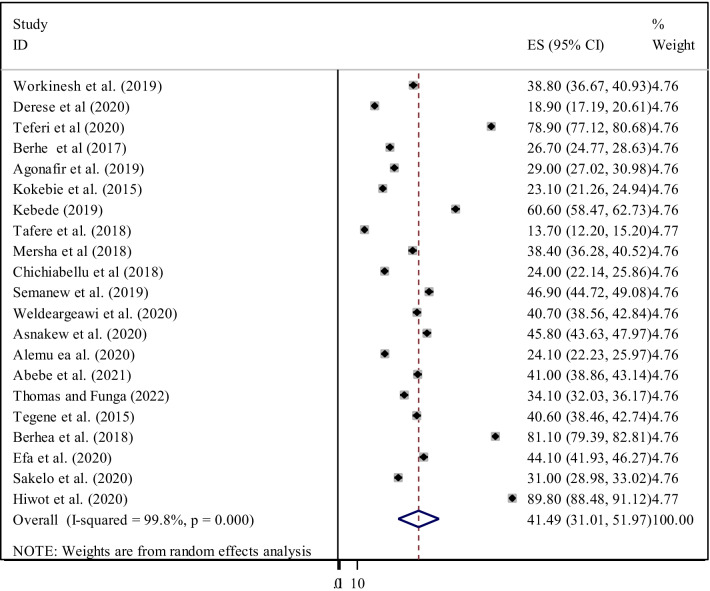
Forest plot of the pooled prevalence of practice of essential newborn care among women in Ethiopia

The pooled prevalence of adequate level of ENC knowledge among Ethiopian women was 55.04% (95% CI 43.18, 66.90) (Fig. 2). The pooled estimated national level of ENC practice among women was 41.49% (95% CI 31.01, 51.97) (Fig. 3). Among the nine studies dealing with knowledge of ENC, there was extreme heterogeneity across the studies (I^2^ = 99.8, *p* < 0.001). there was also heterogeneity across the twenty studies included in this meta-analysis to pool the practice of ENC (I^2^ = 99.8, *p* < 0.001). Hence, a random effect meta-analysis model was used to estimate the pooled prevalence of ENC knowledge and practice among women in Ethiopia. Therefore, to identify the source of heterogeneity, subgroup analyses were conducted based on region and study setting. We also performed a sensitivity analysis to identify any outlier studies.

### Subgroup analysis

After realizing the heterogeneity of the studies, subgroup analysis was performed based on the region where the primary studies were done and the study setting. The subgroup analysis by the region showed that the highest pooled prevalence of ENC knowledge was observed in Amhara regional state 68.26% (95% CI 42.88, 93.64), and the lowest pooled in SNNPR 48.43% (95% CI 36.98, 59.89). Besides, the subgroup analysis was done by study setting, indicating that institutional-based studies had a higher pooled prevalence of 58.38% (95% CI 40.29, 76.47) than institutional-based studies 52.37% (95% CI 35.16, 69.57).

The subgroup analysis of ENC practice by region and study setting was also calculated to compare ENC practice across regions of the country and among different study settings. Accordingly, the highest practice of ENC was observed in Tigray regional state 56.85% (95% CI 29.89, 83.82) followed by Amhara region 46.65% (95% CI 20.04, 73.26). Whereas, the lowest practice was observed in Oromia regional state 31.49% (95% CI 6.80, 56.19). The highest magnitude of ENC practice was observed among community-based studies 45.80% (95% CI 22.94, 68.66) (Table 2).

**Table 2:** Subgroup analysis of knowledge and practice of essential newborn care among women in Ethiopia

Variables	Characteristics	Included studies	Number of study participants	Prevalence (95% CI)	I^2^ (%), *P*-value
For knowledge of ENC					
Region	SNNP	3	1653	48.43 (36.98–59.89)	99.8, < 0.001
	Tigray	2	752	58.25(14.85, 101.67)	99.9, < 0.001
	A. A	1	512	39.80 (37.66, 41.94)	–
	Amhara	2	600	68.26 (42.88, 93.64)	99.7, < 0.001
	Harar	1	266	57.20 (55.04, 59.36)	–
Study setting	Community	5	2405	52.37(35.16, 69.57)	99.7, < 0.001
	Institutional	4	1378	58.38(40.29, 76.47)	99.7, < 0.001
Overall		9	3783	55.04 (43.18, 66.90)	99.7, < 0.001
Practice of ENC					
Region	SNNPR	7	3849	31.64 (26.76, 36.52)	97.6, < 0.001
	Amhara	6	3377	46.65 (20.04, 73.26)	99.9, < 0.001
	Tigray	4	1902	56.85 (29.89, 83.82)	99.9, < 0.001
	Oromia	2	1054	31.49 (6.80, 56.19)	99.7, < 0.001
	A. A	1	576	38.80 (36.67, 40.93)	–
	Benishangul	1	539	40.60 (38.46, 42.74)	–
Study setting	Community	14	7883	39.34 (28.34, 50.33)	99.8, < 0.001
	Institution	7	3414	45.80 (22.94, 68.66)	99.9, < 0.001
Overall		21	11,297	41.49 (31.01, 51.97)	99.8, < 0.001

### Sensitivity analysis

To identify any outlier reports that could affect the pooled prevalence of ENC knowledge and practice by giving a wide confidence interval and variance instability, we checked each study’s sensitivity. The result of the sensitivity analysis indicated that the finding did not rely on a particular study and no study detected that was significantly affected the pooled prevalence of ENC knowledge and practice (Table 3).

**Table 3:** Sensitivity analysis of the prevalence of level knowledge and practice of essential newborn care among women in Ethiopia

Study omitted	Prevalence (%)	95% CI
For knowledge of ENC		
Berhan et al. [19]	56.94	44.41, 69.47
Kebede et al. [20]	55.00	41.67, 68.34
Misgna et al. [21]	51.86	40.47, 63.26
Abebe et al. [22]	55.66	42.47, 68.86
Thomas and Funga [23]	57.20	44.90, 69.50
Zewde [24]	54.76	41.41, 68.12
Berhea et al. [25]	57.40	45.31, 69.50
Mersha et al. [26]	54.71	41.36, 68.07
Yisak et al. [43]	51.76	40.64, 62.88
For practice of ENC		
Workinesh et al. [27]	41.62	30.67, 52.58
Derese et al. [28]	42.62	31.90, 53.33
Teferi et al. [29]	39.62	29.28, 49.95
Berhe et al. [49]	42.23	31.35, 53.11
Agonafir [31]	42.11	31.21, 53.02
Kokebie et al. [32]	42.41	31.58, 53.23
Kebede [20]	40.53	29.68, 51.38
Tafere et al. [33]	42.88	32.43, 53.23
Mersha et al. [34]	41.64	30.69, 52.60
Chichiabellu et al. [35]	42.36	31.52, 53.20
Semanew et al. [36]	41.22	30.27, 52.16
Weldeargeawi et al. [37]	41.53	30.57, 52.48
Asnakew et al. [38]	41.27	30.32, 52.22
Alemu et al. [39]	42.36	31.52, 53.20
Abebe et al. [22]	41.51	30.55, 52.47
Thomas and Funga [23]	41.86	30.92, 52.80
Tegene et al. [40]	41.53	30.58, 52.49
Berhea et al. [25]	39.51	29.32, 49.69
Efa et al. [41]	41.36	30.40, 52.31
Sakelo et al. [42]	42.01	31.09, 52.93
Yisak et al. [43]	39.07	30.40, 47.74

### Publication bias

**Fig. 4:**
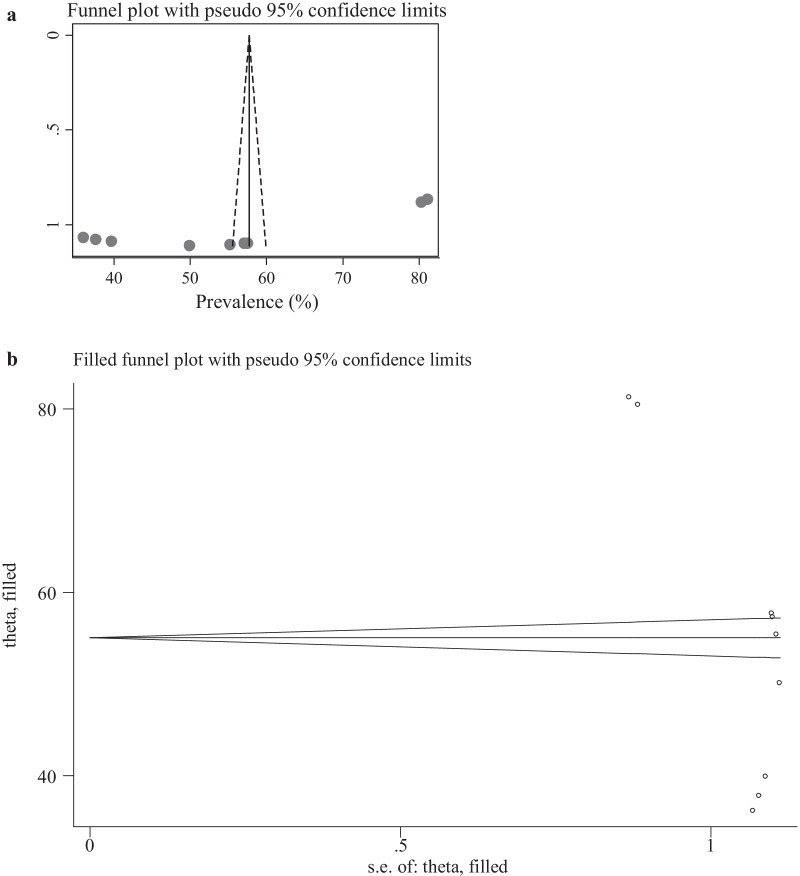
**a** Funnel plot to test the publication bias of 9 knowledge assessing studies. **b** Result of trim and fill analysis for adjusting publication bias

**Fig. 5:**
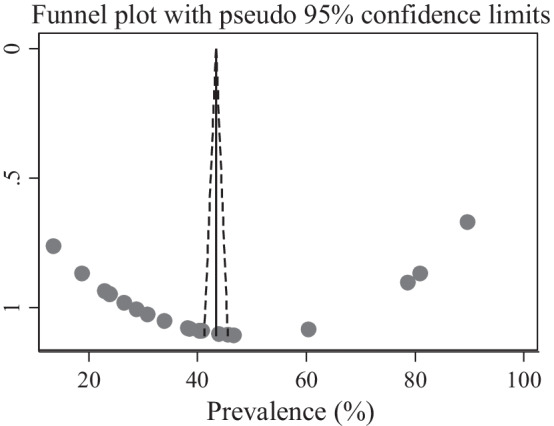
Funnel plot to test the publication bias of included 21 studies to assess practice of essential newborn care among women in Ethiopia

Publication bias among the included studies to assess ENC knowledge was checked using visual inspection of funnel plot and Egger’s test. Publication bias was observed according to Egger’s test (P = 0.028) and the shape of funnel plots was asymmetrical (Fig. 4a). The Duval and Tweedie non-parametric trim and fill analysis was conducted to correct publication bias among the eight studies reporting on ENC knowledge. However, no trimming was performed meaning that the data was unchanged (Fig. 4b). We also assess the presence of publication bias among twenty ENC practice assessing studies. However, publication bias was not observed as evidenced by the symmetrical shape of the funnel plot (Fig. 5) and Eggers’s regression test (P = 0.422).

### Factors affecting knowledge of ENC

This systematic review and meta-analysis noticed that ENC knowledge among women in Ethiopia was significantly associated with the educational status of the women, parity, ANC follow-up, and PNC visit.

**Fig. 6:**
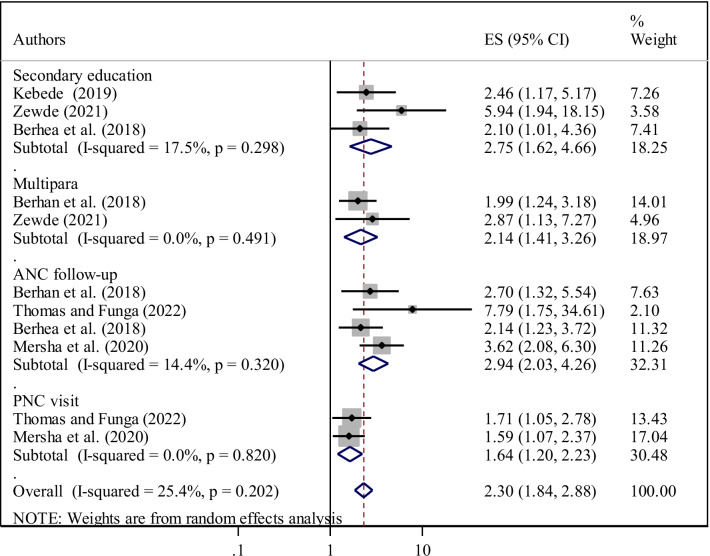
Factors affecting women’s knowledge of essential newborn care among women in Ethiopia

Three primary studies [20, 24, 25] identified that secondary education was significantly associated with ENC knowledge. Women who attended secondary education were nearly three times (AOR = 2.75, 95% CI 1.62, 4.66) more likely to know about ENC than women who had no formal education. The heterogeneity test indicated I^2^ = 17.5%, *P* = 0.29 hence the random-effect model was applicable to yield the pooled odds ratio (Fig. 6).

In this meta-analysis, two studies [19, 24] also reported that being multipara was strongly associated with ENC knowledge. Compared with those who were primipara, multipara women were two times (AOR = 2.14, 95% CI 1.41, 3.26) more likely to know about ENC. Due to the absence of heterogeneity between the included studies (I^2^ = 0.0%, *P* = 0.49) fixed-effect model was used for analysis (Fig. 6).

ANC follow-up was also the single most predictor of ENC knowledge, as highlighted in four primary studies [19, 23, 25, 26]. Women who have been attended ANC follow-up had nearly three times (AOR = 2.94; 95% CI 2.03, 4.26) higher odds to receive an adequate level of knowledge on ENC than counterparts. The heterogeneity test showed an I^2^ value of 14.4%, *P* = 0.32 hence, we used the random-effect model for analysis (Fig. 6).

Once more, as evidenced by two primary articles [23, 26], women who have been attended PNC visit nearly two times (AOR = 1.64, 95% CI 1.20, 2.23) increased the odds of ENC knowledge compared to those women who have not attended PNC visit. Heterogeneity was not observed across the studies (I^2^ = 0.0%, P = 0.82) (Fig. 6).

### Factors affecting the practice of ENC

Maternal education, residency, attending pregnant mothers meeting, antenatal (ANC) follow-up, place of birth, advised on ENC by health care providers during labor and delivery, postnatal follow-up, and knowledge on ENC were found to be statistically significant with the practice of ENC among women in Ethiopia.

**Fig. 7:**
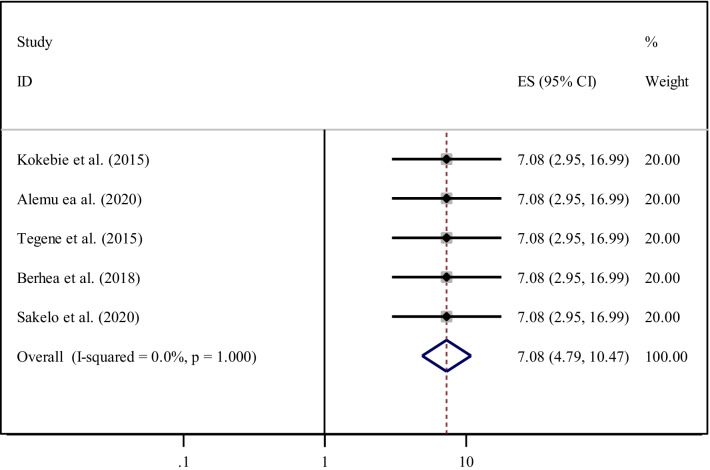
Forest plot showing the pooled odds ratio of the association between primary education and above and practice among women in Ethiopia

According to the current meta-analysis, five studies [25, 32, 39, 40, 42] reported that women who attended primary education and above were significantly associated with the practice of ENC. The pooled odds ratio indicated that women who attended primary education and above were seven times (AOR = 7.08, 95% CI 4.79, 10.47) more likely to practiced ENC. Since heterogeneity effect was not evident (I^2^ = 0.0%, *P* = 1.00) the fixed-effect model was used for analysis (Fig. 7).

**Fig. 8:**
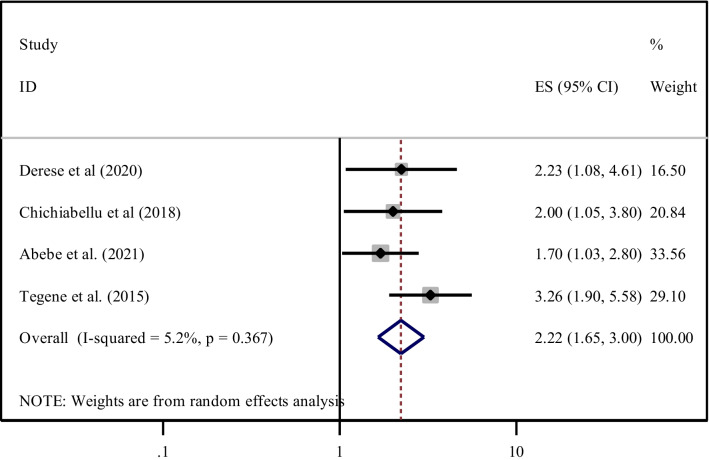
Forest plot showing the pooled odds ratio of the association between urban residency and practice among women in Ethiopia

Four studies [22, 28, 35, 40] also indicated that urban residency was positively associated with ENC practice. The overall estimates revealed that women who have lived in urban areas were 2.2 times (AOR = 2.22, 95% CI 1.65, 3.00) more likely to exercised ENC practice compared to their counterparts. There is minimal heterogeneity across the studies (I^2^ = 5.2%, *P* = 0.36) hence, a fixed-model was used to pool the odds ratio (Fig. 8).

**Fig. 9:**
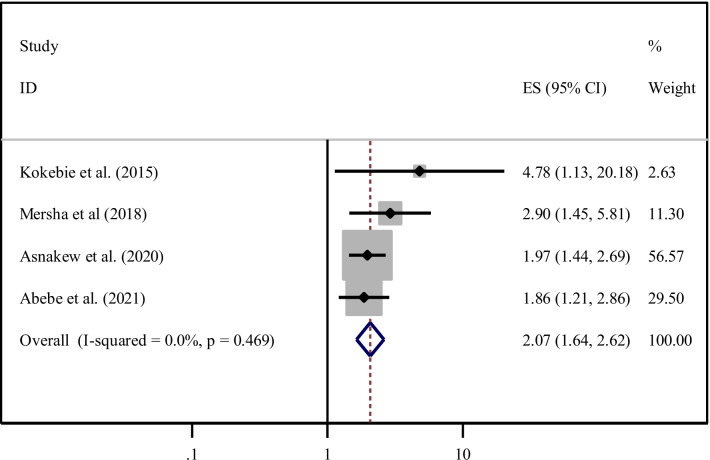
Forest plot showing the pooled odds ratio of the association between attending of pregnant mothers monthly meeting and practice among women in Ethiopia

Attending pregnant mother’s monthly meetings was another significant factor associated with the practice of ENC [22, 32, 34, 38]. Women who have been attended the pregnant mothers monthly meeting were two times (AOR = 2.07, 95% CI 1.64, 2.62) increased the odds of practicing ENC compared to their counterparts. Due to the absence of heterogeneity across the studies (I^2^ = 0.0%, *P* = 0.469), the random-effect model was used to estimate the pooled odds ratio (Fig. 9).

**Fig. 10:**
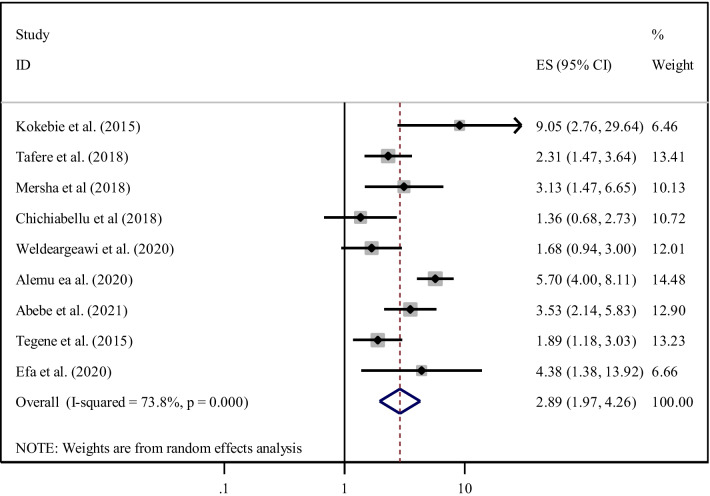
Forest plot showing the pooled odds ratio of the association between ANC follow-up and practice among women in Ethiopia

This meta-analysis reported that ANC follow-up was a significant predictor for ENC practice as evidenced by nine studies [22, 32–35, 37, 39–41]. Women who have attended ANC follow-up during their pregnancies were nearly three times (AOR = 2.89, 95% CI 1.97, 4.26) more likely to practice ENC than those women who have not attended ANC follow-up. Since heterogeneity was observed (I^2^ = 73.8% and p < 0.001), a random effect meta-analysis model was used to determine the association (Fig. 10).

**Fig. 11:**
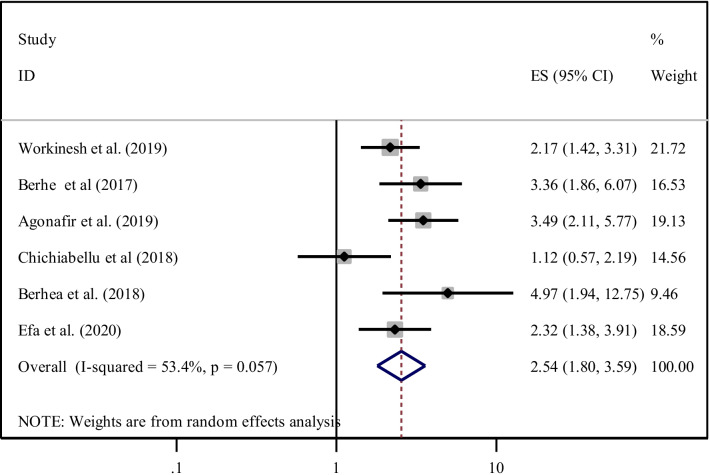
Forest plot showing the pooled odds ratio of the association between advice on essential newborn care during delivery its practice among women in Ethiopia

In the current study, six studies [25, 27, 31, 35, 41, 49] also indicated that women who have been advised about ENC by health care providers during labor and delivery were highly exercised ENC practice. The pooled odds ratio indicated that the odds of ENC practice among women who have been advised on ENC were about 2.54 times higher than women who have not advised about ENC (AOR = 2.54, 95% CI 1.80, 3.59). Since heterogeneity was exhibited (I^2^ = 53.4% and p = 0.057), a random effect meta-analysis model was used to determine the (Fig. 11).

**Fig. 12:**
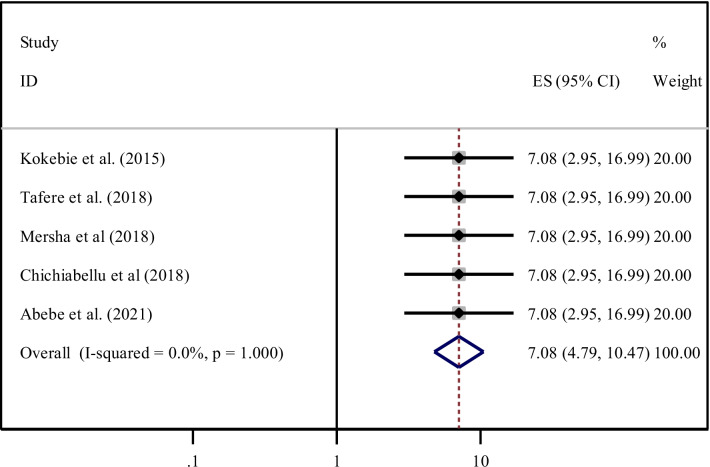
Forest plot showing the pooled odds ratio of the association between PNC follow -up and practice of ENC among women in Ethiopia

Moreover, five studies [22, 32–35] were assessed for the association between PNC follow-up and ENC practiced. Accordingly, women who had immediate PNC follow-up were seven times (AOR = 7.08, 95% CI 4.79, 10.47). increased the odds of newborn care practice as compared to their counterparts. Heterogeneity was not observed across the studies (I^2^ = 0.0%, P = 1.00) (Fig. 12).

**Fig. 13:**
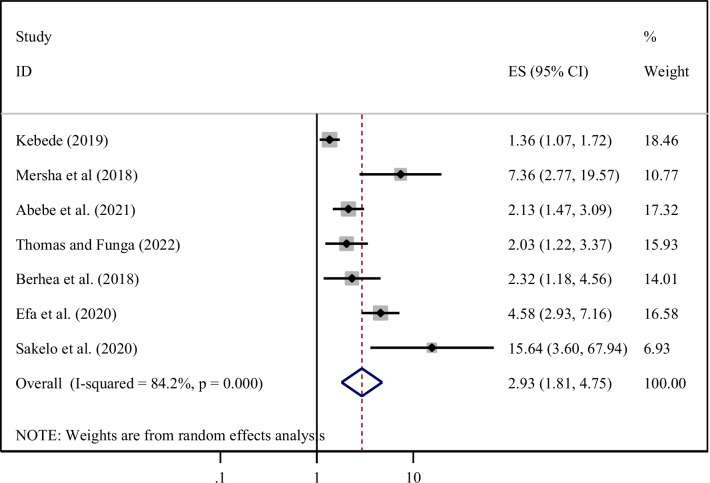
Forest plot showing the pooled odds ratio of the association between knowledge and practice of ENC among women in Ethiopia

Finally, seven studies [20, 22, 23, 25, 34, 41, 42] revealed that women who had good knowledge of ENC were nearly three times (AOR = 2.93; 95% CI 1.81, 4.75) more likely to practice ENC practice when compared with those women who had poor knowledge of ENC. The heterogeneity test showed that (I^2^ = 82.4%, *P* < 0.001) hence, the random-effect model was used for analysis (Fig. 13).

## Discussion

The authors performed this systematic review and meta-analysis to estimate the pooled prevalence of ENC level of knowledge and practice and its associated factors among women in Ethiopia. In developing countries, like Ethiopia conducting such a kind of study will be paramount as input for program planners and policymakers working in the area of achieving sustainable development goal (SDG) three related to newborn health. Accordingly, the pooled prevalence of adequate level of ENC knowledge among Ethiopian women was 55.04% (95% CI 43.18, 66.90). The overall prevalence reported in this meta-analysis is consistent with a survey study conducted in Pakistan (57%) [50], India (60.0%) [51], Himalayas (51%) [52], Ghana (62.2%) [53]. However, the finding was lower than a study conducted in Bangladesh (88.7%) [54]. The possible explanation might be due to the low socio-economic status, difference in infrastructure, accessibility of the health care system, inefficient promotion of ENC on media, the low commitment given to community-based newborn care. This review suggests that the awareness-creating approaches of ENC were insufficient. Hence, involving various stakeholders, governmental and non-governmental organizations regarding ENC is our future insight to increase women’s knowledge.

The current systematic review and meta-analysis also endeavored to determine the pooled prevalence of ENC practice. Accordingly, 41.49% (95% CI 31.01, 51.97) of Ethiopian women have practiced ENC. The finding was inlined with a study conducted in Nepal 37.2% [55]. On the other hand, our finding was lower than the national prevalence of Pakistan (57.1%) [50], Himalayas (66.2%) [52], Ghana (72.8%) [53]. The possible explanation might be due to differences in socioeconomic status and socio-cultural between the study countries, the study period, and the study setting. Besides, variation in health care service coverage may have a direct influence on women awareness and information about the practice of ENC. It is also explained by poor strategies and guiding principles and low media coverage for ENC in the country.

Women with secondary education and above were found to have an adequate level of knowledge about ENC than those who had no formal education. Our finding was similar to studies conducted in India which reported that there was an association between ENC knowledge and educational status [56–58]. Another study conducted in Iran [59] and Himalayas [52] also mentioned educational status as a positive predictor for adequate ENC knowledge. As the educational status of women increases; their health-seeking behavior concerning ENC will also expand. as a result, they become ready to know about their newborn health status, and risk factors leading to ill health. Besides, more educated women might have better access or be more persuaded to acquire evidence sources about their newborn wellbeing and have better newborn care management plans. Moreover, having adequate knowledge of ENC helps to easily understand the newborn complication associated with lack of ENC.

The current systematic review also reported that ENC knowledge increases with parity. Multipara women had an increased level of knowledge on newborn care. The finding is supported by study findings conducted in six low- and middle-income countries [60]. Findings might be due to women’s having prior experience of delivery can get information about essential newborn care during their previous ANC, Delivery, PNC, and immunization periods and this can help to improve women’s knowledge level on newborn care and inspire them to exercise ENC practice more.

In terms of health care utilization, women who had ANC visits during pregnancy were possessed an adequate level of ENC knowledge compared to their counterparts. Finding from Himalayas [52], Sri Lanka [61], and Kenya also supported the current finding. During ANC service utilization women were counseled about birth preparedness and complication readiness (BPCR) plan which may increase the awareness of women regarding ENC [31, 32]. as BPCR is one of the 12 basic WHO recommendations for increasing the use of skilled maternity care and minimizing serious obstetric and neonatal complications by the well-timed use of facility care [62]. Also, ANC creates an opportunity for the women to communicate with the health care provider on the issue of newborn health during pregnancy, childbirth, and in the post-natal period.

Similarly, women who had postnatal follow-up also had a good level of knowledge on ENC. The finding was inlined with studies done in India [63], Himalayas [52], and South Sudan [64]. The possible justification could be women who attended PNC follow-up, might have the chance of getting information about the importance of ENC from health professionals.

According to this review, maternal educational status was significantly associated with ENC practice. The finding of this review study was comparable with a study done in Uganda, Ghana, and India [65, 66], Himalayas [52], Iran [67], and Pakistan [50]. This finding might be because educating the women were being a community and political concern in the world. Women’s education is a universal agenda to be implemented as a prospective countries program and is playing a great role to have good newborn care practices. In addition, an educated mothers may have a better understanding of the ENC practices, and they had the chance of accessing information from their schools and by reading books, and using social media related to ENC. Furthermore, a highly educated mothers could have an improved insight or awareness about the importance of implementing and the risk of not implementing ENC.

In this meta-analysis, women who live in urban areas were more likely to practice ENC than rural women. The finding was supported by a study done in Pakistan [50]. The finding might be since women with urban residency had higher access to information through media coverage that might sustenance them in decision making concerning healthy behaviors of newborn health. Besides, urban residency may also increase access to health services and adequate knowledge secondary to better educational status. Moreover, urban residence women may have easy access to healthcare services and better infrastructure which ultimately leads to better practice of ENC.

In the current review, women attending pregnant mother’s monthly meetings were highly practiced ENC compared to their counterparts. The possible explanation could be the health care providers could provide health education and promotion about essential newborn care practices during the meeting. The Ethiopian Ministry of Health initiated a community-based monthly meeting intervention for pregnant mothers to increase awareness of obstetric dangers and the use of institutional skilled maternal health care services [68]. These monthly meetings are led by the community health extension workers (HEWs) and midwives [68–70]. These monthly meetings scale up uptake of the knowledge and practice of ENC by promoting a sense of peer support among women to pursue adequate ANC, planning for delivery at a health facility, following through with appropriate PNC, and promoting exclusive breastfeeding [68]. Hence, health care providers in the community and health facilities must work actively to initiate and strengthen these conferences.

The current review also revealed women’s practice of ENC was enhanced by antenatal care service utilization. Women who attended antenatal care visits had an increased chance of ENC practice as compared to those who had no ANC visit. The findings were consistent with a study conducted in Ghana [71], Uganda [72], Tanzania [73], Nepal [74], and Himalayas [52]. This might be due to women who attended ANC have the chance of receiving information concerning the components as well as the merit of practicing ENC from the health professionals. Besides, women who visited ANC would get counseling on the importance of giving birth by skilled birth attendants, and institutional delivery which is believed to scale up their knowledge and practice of ENC.

Moreover, our review noticed that getting advice on ENC during labor and delivery was positively linked with the practice of ENC. Those women who had gotten advice on the importance of ENC practice in the time of labor and delivery were nearly three times increase the odds of exercising ENC practice. One protentional reason might be women who had advised the care that must be provided to their newcomer (newborn) by a health care professionals would enhance their awareness and practice of essential newborn care. Once more skilled birth attendant’s advice on the obstetric world has been believed as a key factor prompting the healthy outcome of both the mother and newborn [75, 76].

This meta-analysis also showed that exercising ENC increases with the frequency of post-natal follow-up. Women who had one and/or more PNC follow-up had an adequate level of knowledge regarding ENC. Findings from Nepal [74], Bachauli and Khairahani [77], and Himalayas [52] also emphasized that PNC follow-up had a positive impact on women’s level of knowledge about ENC. This could be women who had PNC follow-ups were counseled about essential newborn care by health care providers and health extension workers which may increase their practice. Community health workers may also advise about the ENC care practice during immediate PNC visits.

Lastly, women with a good knowledge scores of ENC were positively correlated with practice of ENC. Our finding was consistent with study findings from Nepal [78], Chitwan [79], and Himalayas [52]. The possible reason might be accumulated experience and awareness about components of ENC would help mothers to put essential newborn care into practice. Besides, women with an adequate level of knowledge regarding ENC were more in a position to anticipate costs associated with not practicing ENC.

Behind its multilevel substantial importance, the current systematic review and meta-analysis was not ended without limitation. First, due to the cross-sectional nature of all included primary studies the outcome variable may be affected by confounding variables like a misconception, accessibility of care. Second, studies with a small sample size may negatively affect the national estimation of the ENC knowledge and practice of women. Lastly, only six regional stats and one administrative town were involved due to limited studies.

## Conclusion and recommendation

The current systematic review and meta-analysis findings reported that almost half (55.04%) and only 41.49% of the women have an adequate level of knowledge and practice of ENC respectively. Educational status, parity, ANC, and PNC visits were predictors of knowledge of ENC whereas educational status, residency, ANC visit, attending the monthly meetings, advice during delivery, PNC visit, and knowledge were statistically significant with the practice of ENC. Therefore, Improvement of ENC through the provision of community-oriented awareness creation forum and counseling and promotion of ANC and PNC care follow up, education on essential newborn care, and neonatal danger signs to all pregnant and post-natal women are very important.

## Supplementary Information

**Additional file 1.** Searching strategy for knowledge and practice of essential newborn care and associated factors among women in Ethiopia.

**Additional file 2.** Newcastle–Ottawa Quality Assessment Scale for cross-sectional studies to assess knowledge and practice of essential newborn care among women in Ethiopia.

## Data Availability

The data set that used in this review are available upon a reasonable request to the corresponding author.

## Abbreviations

ANC: Antenatal care
PNC: Postnatal care
ENC: Essential newborn care
