# Supplementary material for: Knowledge and practice of essential newborn care and associated factors among women in Ethiopia: systematic review and meta-analysis
Source: Reprod Health. 2022 Aug 4;19:172. doi: 10.1186/s12978-022-01480-0 (PMC9351089; doi:10.1186/s12978-022-01480-0)
Supplement: Supplementary file 1 — Additional file 1. Searching strategy for knowledge and practice of essential newborn care and associated factors among women in Ethiopia. [file 12978_2022_1480_MOESM1_ESM.docx]

**Additional file 1:** Searching strategy for knowledge and practice of essential newborn care and associated factors among women in Ethiopia

| Databases | Searching terms | Number of studies |
| --- | --- | --- |
| PubMed | "Knowledge"[MeSH Terms] OR “knowledge” [All Fields] AND "practice" [MeSH Terms] OR “practice” [All Fields] AND “essential” [MeSH Terms] OR “essential” [All Fields] AND "newborn" [MeSH Terms] OR "newborn" [All Fields] AND “care” [MeSH Terms] OR “care” [All Fields] AND “associated factors” [MeSH Terms] OR “associated factors” [All Fields] OR “predictors” [MeSH Term] OR “predictors” [All Fields] OR “determinants” [MeSH Terms] OR “determinants” [All Fields] AND "women" [MeSH Terms] AND “Ethiopia” [All Fields] | 288 |
| Google scholar | “Knowledge” AND “practice” AND “essential” AND “newborn care” AND “associated factors” AND “women” AND “Ethiopia” | 740 |
| HINARI | “Essential” AND “newborn care “AND “knowledge” AND “practice” AND “associated factors “AND “women” AND “Ethiopia” | 241 |
| Others databases |  | 6 |
| Total retrieved |  | 1275 |
| **Included** |  | **25** |
