# Supplementary material for: Knowledge and practice of essential newborn care and associated factors among women in Ethiopia: systematic review and meta-analysis
Source: Reprod Health. 2022 Aug 4;19:172. doi: 10.1186/s12978-022-01480-0 (PMC9351089; doi:10.1186/s12978-022-01480-0)
Supplement: Supplementary file 2 — Additional file 2. Newcastle–Ottawa Quality Assessment Scale for cross-sectional studies to assess knowledge and practice of essential newborn care among women in Ethiopia. [file 12978_2022_1480_MOESM2_ESM.docx]

**Additional file 2:** Newcastle-Ottawa Quality Assessment Scale for cross-sectional studies to assess knowledge and practice of essential newborn care among women in Ethiopia

| Authors | Representativeness | Sample size | None-responders | Ascertainment | comparability | outcome | Quality score |
| --- | --- | --- | --- | --- | --- | --- | --- |
| Berhan et al. (2018) | 2 | 2 | 1 | 1 | 1 | 1 | 8 |
| Kebede et al. (2019) | 1 | 1 | 1 | 2 | 1 | 1 | 7 |
| Misgana et al. (2016) | 1 | 1 | 1 | 2 | 1 | 1 | 7 |
| Abebe et al. (2021) | 2 | 2 | 1 | 1 | 1 | 1 | 9 |
| Thomas (2018) | 1 | 1 | 1 | 1 | 2 | 1 | 7 |
| Gossaye (2021) | 1 | 1 | 1 | 1 | 2 | 1 | 7 |
| Berhea et al. (2018) | 2 | 1 | 1 | 2 | 1 | 1 | 8 |
| Mersha et al. (2020) | 2 | 2 | 1 | 1 | 2 | 1 | 9 |
| Daba et al (2018) | 2 | 2 | 1 | 1 | 1 | 1 | 8 |
| Derese et al. (2020) | 2 | 2 | 1 | 1 | 2 | 1 | 9 |
| Teferi et al. (2020) | 1 | 2 | 1 | 1 | 1 | 1 | 7 |
| Berhe et al. (2017) | 1 | 2 | 1 | 1 | 1 | 1 | 7 |
| Agonafir (2019) | 2 | 1 | 1 | 1 | 2 | 1 | 8 |
| Kokebie et al. (2015) | 2 | 2 | 1 | 1 | 1 | 1 | 8 |
| Tafere et al. (2018) | 2 | 2 | 1 | 2 | 1 | 1 | 9 |
| Mersha et al. (2018) | 2 | 2 | 1 | 2 | 1 | 1 | 9 |
| Chichiabellu et al. (2018) | 2 | 2 | 1 | 1 | 1 | 1 | 8 |
| Semanew et al. (2019) | 2 | 1 | 1 | 1 | 1 | 1 | 7 |
| Weldearegawi et al (2020) | 2 | 2 | 1 | 1 | 1 | 1 | 8 |
| Asnakew et al. (2020) | 2 | 2 | 1 | 1 | 2 | 1 | 9 |
| Alemu et al. (2020) | 2 | 2 | 1 | 2 | 1 | 1 | 9 |
| Tegegne et al. (2015) | 2 | 2 | 1 | 1 | 2 | 1 | 9 |
| Efa et al. (2020) | 2 | 2 | 1 | 1 | 1 | 1 | 8 |
| Sakelo et al. 2020 | 1 | 2 | 1 | 2 | 1 | 1 | 7 |
| Hiwot et al. (2020) | 1 | 1 | 1 | 2 | 1 | 1 | 7 |

Interpretation of the score

Very Good Studies: 9-10 points

Good Studies: 7-8 points

Satisfactory Studies: 5-6 points

Unsatisfactory Studies: 0 to 4 points
